# Three-dimensionally printable hollow silica nanoparticles for subambient passive cooling

**DOI:** 10.1515/nanoph-2023-0603

**Published:** 2024-01-23

**Authors:** Su-Jin Park, Seok-Beom Seo, Jiyun Shim, Seok Jin Hong, Gumin Kang, Hyungduk Ko, Sunho Jeong, Sun-Kyung Kim

**Affiliations:** Department of Applied Physics, Kyung Hee University, Yongin-si, Gyeonggi-do 17104, Republic of Korea; Department of Advanced Material Engineering for Information & Electronics, Kyung Hee University, Yongin-si, Gyeonggi-do 17104, Republic of Korea; Nanophotonics Research Center, Korea Institute of Science and Technology, Seoul 02792, Republic of Korea

**Keywords:** passive cooling, hollow nanoparticle, three-dimensional printing, additive manufacturing, subambient cooling, solar energy application

## Abstract

Solar reflectance and thermal emissivity are critical benchmarks for evaluating the effectiveness of passive cooling strategies. The integration of three-dimensional (3D) printing techniques with passive cooling materials enables local thermal management of multifaceted objects, offering opportunities for unexplored energy-saving applications. For example, conformal printing of cooling materials can mitigate solar absorption caused by the top metal electrodes in solar cells, thereby improving their efficiency and lifetime. In this study, we report the synthesis of 3D printable hollow silica nanoparticles (HSNPs) designed to induce subambient cooling performance under daylight conditions. HSNPs with diameters of 400–700 nm and silica shell thicknesses of approximately 100 nm were synthesized using an *in-situ* sol–gel emulsion method. Subsequently, these HSNPs were formulated into printable pastes by carefully selecting the mixture concentration and molecular weight of polyvinylpyrrolidone (PVP). The PVP-linked HSNPs exhibited a solar (0.3–2.5 μm) reflectivity of 0.98 and a thermal (8–13 μm) emissivity of 0.93. In contrast to a single silica nanoparticle (NP), the scattering analysis of a single HSNP revealed a distinctive scattering distribution characterized by amplified backward scattering and suppressed forward scattering. In outdoor daytime experiments, the HSNP-printed sample led to the subambient cooling of a dielectric substrate, surpassing the cooling performance of reference materials such as silica NPs, silver pastes, and commercial white plastics and paints.

## 1 Introduction

The device temperature is critical in determining the overall performance and lifetime of semiconductor optoelectronic devices such as solar cells and light-emitting diodes in displays [1]. For example, even a modest reduction in the operating temperature of solar cells by 30 °C at the p/n junction can result in a significant increase in quantum efficiency of more than 5 % [2], [3]. Given the intrinsic constraints imposed by thermal effects, an efficient and cost-effective cooling strategy is needed as a viable and transformative measure to overcome the device progress limitations [4], [5].

Radiative cooling has attracted significant attention because of its passive performance [[Bibr j_nanoph-2023-0603_ref_006] [Bibr j_nanoph-2023-0603_ref_007] [Bibr j_nanoph-2023-0603_ref_008], adaptability to various form factors [[Bibr j_nanoph-2023-0603_ref_010] [Bibr j_nanoph-2023-0603_ref_011] [Bibr j_nanoph-2023-0603_ref_012] [Bibr j_nanoph-2023-0603_ref_013] [Bibr j_nanoph-2023-0603_ref_014] [Bibr j_nanoph-2023-0603_ref_015], and seamless integration with other heat transfer mechanisms, including conduction and evaporation [11], [17]. Owing to the energy-saving properties of radiative cooling technology, diverse applications have been demonstrated, including cooling textiles [18], bioinspired durable cooling polymers [19], radiative/evaporative hybrid coolers [20], and quantum-dot-based color coolers [21]. Furthermore, their optical transparency, particularly when visibly transparent oxides and polymers are used, opens possibilities for applications in windows [22] and solar panels [[Bibr j_nanoph-2023-0603_ref_023] [Bibr j_nanoph-2023-0603_ref_024] [Bibr j_nanoph-2023-0603_ref_025]. For solar panels, ideal passive cooling solutions should exhibit transmission within the bandgap of solar cells, reflection from the bandgap within the solar spectrum, and absorption and emission characteristics within the thermal radiation spectrum [27], [28]. Various efforts have been made to integrate such transparent radiative coolers into solar panels, either by incorporating them into the glass cover of solar panels [23], [24] or on the surface of solar cells [25], [26]. In this study, we focused on interdigitated electrodes integrated on the top surface of solar cells. Conventionally, these electrodes are fabricated using Ag paste, which is known for its high solar absorption and limited thermal radiation properties, posing challenges for effective passive cooling [29]. Furthermore, they frequently cause visual discomfort owing to specular reflection, particularly in urban settings [30]. To address these challenges, we developed printable, antiglare, and passive cooling materials using nanoparticles (NPs) interconnected with polyvinylpyrrolidone (PVP) [31], [32].

Recently, Park et al. reported a passive cooling material composed of hollow silica microspheres formed by silica NP aggregation [33]. The developed material exhibits high solar reflectance and thermal emissivity. However, the fabrication process involves the use of sacrificial polymer templates, necessitating high-temperature (550 °C) conditions for a post-hoc calcination process, which hinders the implementation of additive manufacturing techniques. In contrast, Zhou et al. reported a passive cooling material derived from a polymer fused with silica NPs, demonstrating its suitability for three-dimensional (3D) printing applications [34]. This material exhibited a reflectivity of 0.96 across a wavelength range of 0.3–2.0 μm and a thermal emissivity of 0.9 in the atmospheric window (8–13 μm). In outdoor daylight experiments, the polymer-based film reduced the temperature by 6.1 °C when compared to the ambient temperature. However, to form an inner porous morphology, a high-temperature (115 °C) environment and a chemical washing process are required.

In contrast to previous studies, we used an *in-situ* sol–gel emulsion method under ambient conditions to synthesize hollow silica NPs (HSNPs) with diameters ranging from 400 to 700 nm and a silica shell thickness of approximately 100 nm. The HSNPs’ hollow morphology increases backward scattering in the solar spectrum, resulting in ultrahigh solar reflectance [35]. In the thermal radiation spectrum (3–30 μm), the HSNPs serve as a near-perfect blackbody by localizing incident light within their silica shell [36], leading to effective radiative cooling performance. These HSNPs were subsequently combined with PVP at precise concentrations and molecular weights (MWs) to produce a 3D printable paste. The optical spectra of the resulting PVP-linked HSNPs were acquired across ultraviolet to mid-infrared wavelengths using a spectrophotometer and a Fourier transform infrared (FTIR) spectrometer. In contrast to silica NPs, we conducted a scattering analysis to elucidate the near-unity solar reflectance of HSNPs. Finally, outdoor experiments were conducted to reveal the HSNP-based sample’s subambient cooling capabilities and to compare its performance with that of reference samples utilizing silica NPs, silver pastes, and commercial white plastics and paints. Rheology and viscosity measurements indicate that the developed materials can be applied to the conformal printing of 3D multifaceted objects, which is beneficial for miniature devices requiring localized thermal regulation, such as thermoelectric engines designed for drones. Furthermore, the application of our technology to large-scale equipment such as wind turbines [37], [38] and outdoor signal relay stations [39], [40] is expected to contribute substantially to the development of a sustainable energy-based society.

## 2 Results and discussion

### 2.1 Concept of 3D printable passive cooling materials

**Figure 1:**
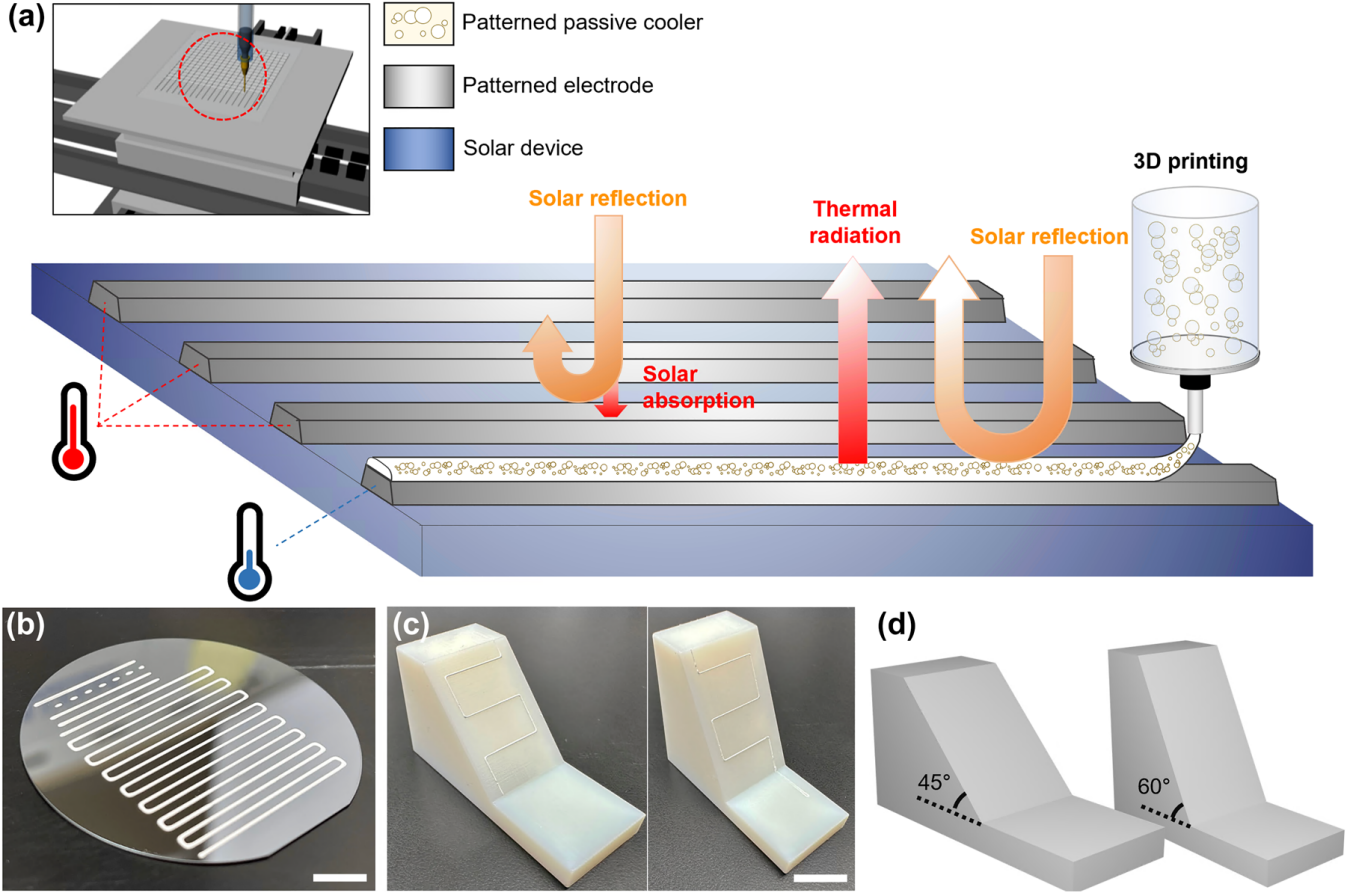
Concept of 3D printable HSNPs. (a) Conceptual schematic illustrating printed HSNPs on a solar device for its passive cooling. (b) Photograph depicting printed lines (a width of approximately 330 μm) composed of HSNPs on a silicone substrate. Scale bar: 1 cm (c) photographs depicting 3D printed lines composed of HSNPs on sloped objects with inclination angles of 45° (left) and 60° (right). Scale bar: 1.5 cm (d) 3D-rendered images of the sloped objects.

We synthesized 3D printable HSNPs to serve as passive cooling materials, with a specific focus on cooling optoelectronic devices, particularly their metal electrodes. Figure 1(a) shows the practical application of printable passive cooling materials on the top electrodes of a solar device. In general, silver-paste-based electrodes suffer from significant absorption within the solar spectrum, resulting in noticeable heating when these devices are exposed to direct sunlight. A viable strategy for eliminating this heating effect is to incorporate passive cooling materials into the upper surface of the electrodes. Specifically, these materials emit thermal radiation within the atmospheric transparent window while simultaneously acting as a mirror in the solar spectrum (0.3–2.5 μm). Stacking of oxide-based NPs is one solution to address these demanding conditions. Notably, our approach utilizes HSNPs to further enhance solar reflectivity and thermal emissivity, and their formulation facilitates the 3D printing process.

The advent of 3D printing has provided a versatile platform for the conformal deposition of target materials along various surfaces of multifaceted objects. With the addition of properly selected binders, HSNPs can be precisely micropatterned on both planar (Figure 1(b)) and sloped surfaces (Figure 1(c) and (d)) using fused deposition modeling (FDM). A serpentine pattern with a defined linewidth of 330 μm, created through the FDM technique, is shown in Figure 1(b). In comparison, Figure 1(c) shows the printed lines on sloped objects with inclinations of 45° and 60°, confirming the efficacy of 3D printing, even on steep slopes. While solar panels feature inclinations ranging from 15°−45°, our deliberate choice of a 60° inclination showcases the robust 3D printability of HSNPs, particularly under more demanding and less conventional conditions.

### 2.2 Solar reflection characteristics

To verify the superior solar reflection of the HSNPs compared with that of their counterpart silica NPs, we employed an integrated ray-wave optics simulation. This simulation technique plays an indispensable role in designing optical devices possessing both microscale and macroscale structural elements [26], [41]. An important example is the design and analysis of antireflective coatings for solar cells, where the upper cover glass has a thickness of up to several millimeters and the surface textures on the solar cell vary in scale from hundreds of nanometers to a few micrometers [26]. This example validates hybrid simulation techniques for addressing complex ray-wave interplay in hierarchically designed structures. In this ray-wave optics simulation, the scattering distribution of each NP was determined through a wave optics simulation and subsequently transferred to a model in a ray optics simulation. Considering the subwavelength scale of NPs and their 3D-packed configuration with thicknesses of up to tens of micrometers, relying solely on either wave or ray optics simulations limits the accurate prediction of the optical characteristics of NPs [42]. By contrast, our integrated ray-wave optics simulation provides both computational accuracy and time efficiency.

**Figure 2:**
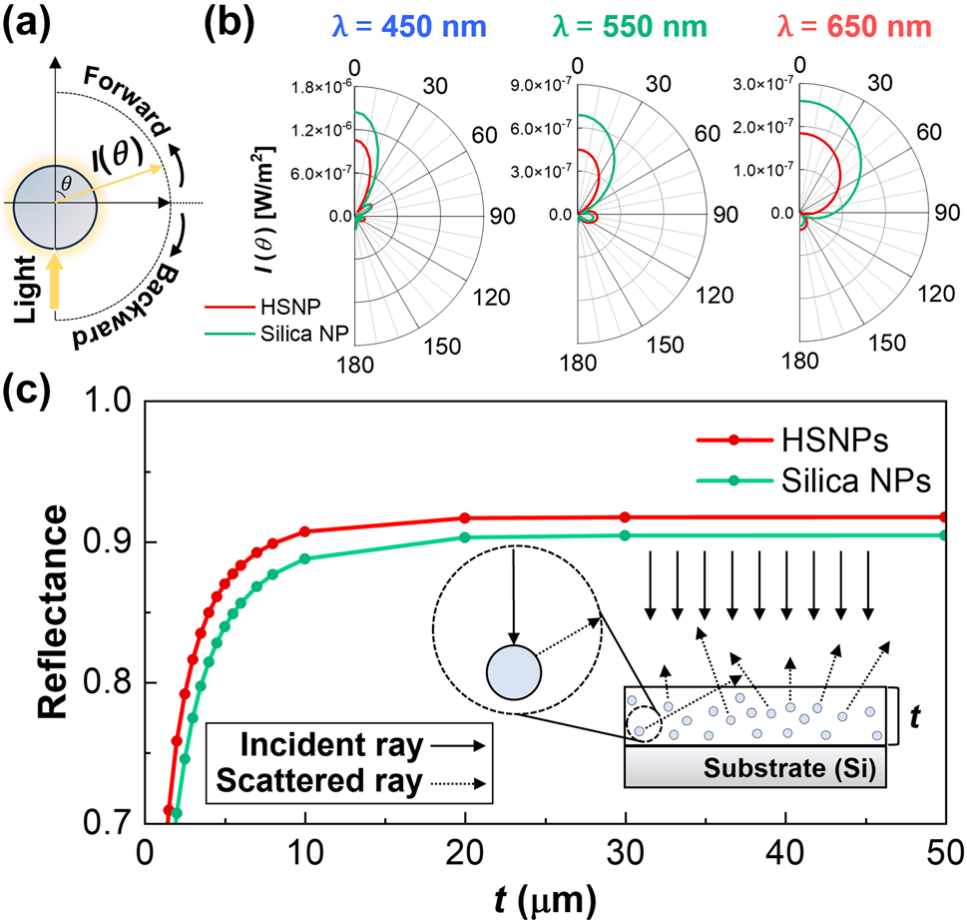
Reflection characteristics of HSNPs. (a) Schematic illustrating the angular scattering distribution of a single NP. (b) Normalized angular scattering distributions of single HSNPs and silica NPs, obtained at three sampled visible wavelengths: 450 (left), 550 (middle), and 650 nm (right). (c) Simulated reflectance values of HSNP- and silica NP-based layers at various thicknesses (*t*). Inset: schematic illustrating the developed integrated ray-wave optics simulation.

The overall reflectance of the stacked NPs was determined by calculating the contributions of the forward and backward scattering distributions of the individual NPs. Assuming that the NPs had a spherical shape, their scattering distributions were defined in terms of the azimuthal angle (*θ*) (Figure 2(a)). Figure 2(b) shows the calculated scattering distributions for single HSNPs and silica NPs with diameters of 500 nm. These distributions were obtained at three primary visible wavelengths (450, 550, and 650 nm). For all the wavelengths considered, while the silica NPs predominantly exhibited forward scattering, the HSNP displayed a pronounced amplitude for backward scattering.

The multipole expansion theory served as the basis for understanding the distinctive scattering distribution of HSNP [43]. In Figure S1(a), the cross-sectional electric field distributions at three visible wavelengths for a single HSNP and silica NP are presented. The hollow morphology of the HSNP impedes the excitation of the electric dipole (ED) in response to incident light (Figure S1(b)). Notably, ED-induced radiation reinforces forward scattering owing to constructive interference with the incident light and destructive interference in the reverse direction [36]. Consequently, the HSNP effectively suppressed forward scattering while enhancing backward scattering (Figure S1(c)). This scattering information was imposed on each NP in a ray-optics simulation (inset, Figure 2(c)). To replicate a close-packed configuration, we set the mean free path of the scattered light to 500 nm, which is equivalent to the diameter of the NPs. Subsequently, we conducted integrated ray-wave optics simulations for both 3D-packed HSNPs and silica NPs, extending their thicknesses up to 50 μm (Figure 2(c)). The reflectance values were derived by averaging the data obtained at the visible wavelengths. Remarkably, the reflectance of HSNPs consistently exceeded that of their silica NP counterparts by an average of 0.013 ± 0.002. This reflectance enhancement is due to the amplified backward scattering characteristics of individual HSNPs.

### 2.3 Synthesis of 3D printable HSNPs

**Figure 3:**
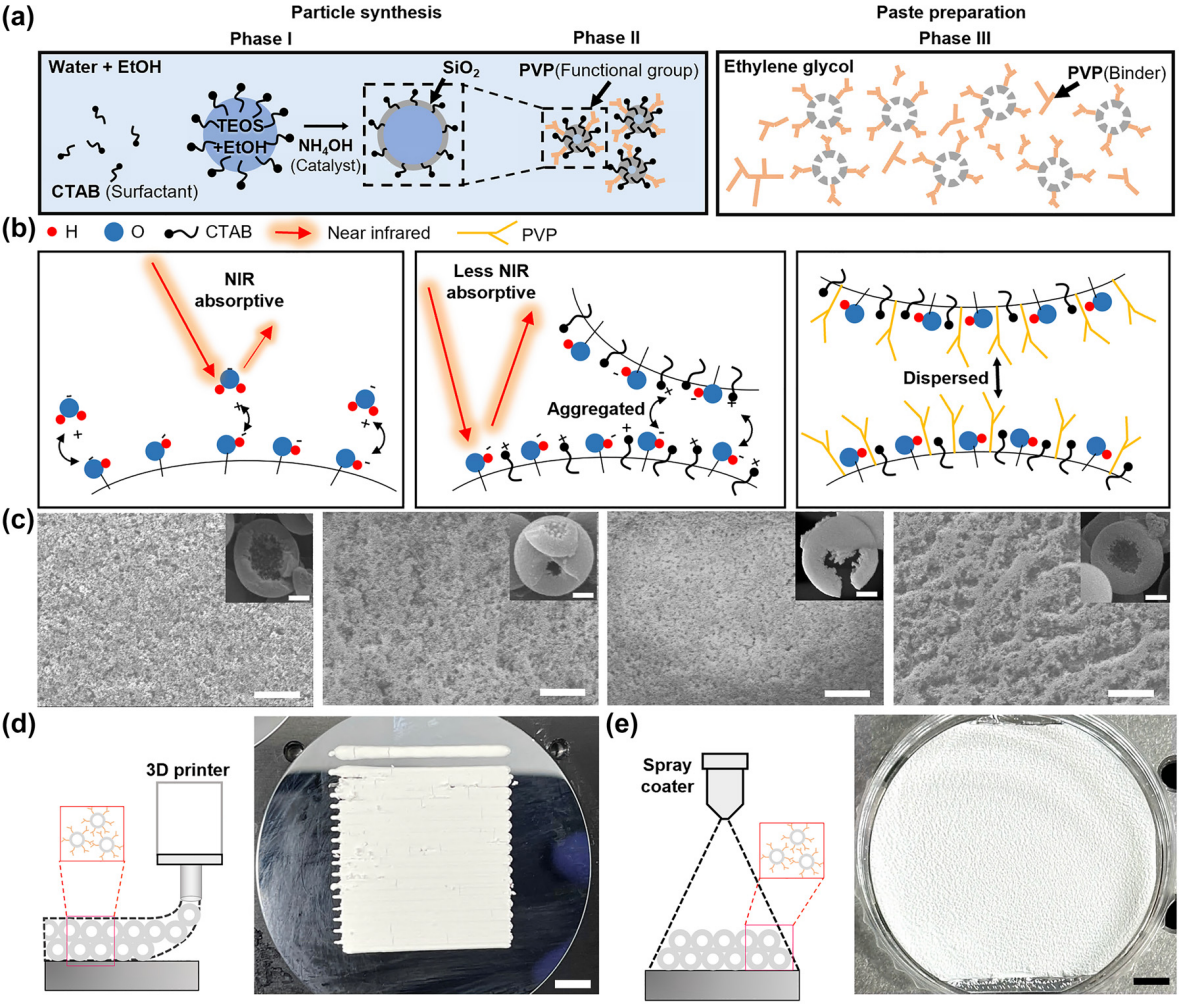
Synthesis of 3D printable HSNPs. (a) Schematics illustrating the synthesis of printable HSNPs: Forming hollow morphology within HSNPs with a CTAB surfactant (Phase I); dispersing HSNPs with a PVP binder (Phase II); formulating printable HSNPs with an additional PVP binder (Phase III). (b) Schematics illustrating the functions of the CTAB surfactant and PVP binder. The CTAB reduces the NIR absorption caused by the hydroxyl group. The PVP prevents HSNPs from being aggregated. (c) SEM images of synthesized HSNPs synthesized under different PVP conditions (MW and weight): (10,000, 25 mg), (55,000, 10 mg), (55,000, 25 mg), and (55,000, 50 mg), from left to right. Scale bars: 50 μm (inset: 200 nm). (d, e) Photographs depicting 3D-printed (d) and spray-coated (e) HSNPs on Si substrates. Insets: schematic of each method. Scale bars: 5 mm.

We employed an *in-situ* sol–gel emulsion method to synthesize HSNPs that serve as 3D printable passive cooling materials, as shown in Figure 3(a). The synthesis process can be categorized into three distinct phases: particle synthesis (Phases I and II) and paste preparation (Phase III). During Phase I, the diameters of the HSNPs were regulated by adjusting the ethanol/water ratio [44]. Phase II included the attachment of PVP molecules to the surfaces of HSNPs, which is a critical step in ensuring their suitability for 3D printing. In Phase III, PVP binders with a mixture of MWs were added to the HSNPs in ethylene glycol. This final step was essential for achieving the desired rheology and viscosity for 3D printing (Figure S2).

Specifically, during the initial Phase I synthesis, cetyltrimethylammonium bromide (CTAB) surfactants served as structural templates for HSNP formation. Within the ethanol solvent, a well-defined interface of micelles distinctly separated the two phases: an internal oil phase encapsulating tetraethyl orthosilicate (TEOS) and an external aqueous phase primarily composed of water. The interface between the oil and aqueous phases promoted the reaction between TEOS and water, thereby catalyzing the formation of silica and initiating the synthesis of ammonia-catalyzed HSNPs under standard temperature and pressure conditions. In Phases II and III, PVP was added and anchored to the surfaces of the HSNPs. This strategic addition of PVP not only enhanced their suitability for 3D printing as a binder but also augmented the repulsive intermolecular forces at play, effectively preventing the undesirable aggregation of HSNPs during the sol–gel reaction.

The use of PVP offers significant benefits in mitigating near-infrared (NIR) absorption, as shown in Figure 3(b). Conventional silica surfaces are susceptible to NIR absorption owing to the adsorption of water molecules, which is caused by the presence of surface hydroxyl groups (left, Figure 3(b)). This phenomenon was confirmed by the increase in NIR reflectance in silica NPs after undergoing high-temperature annealing (Figure S3). The sol–gel emulsion method substantially counters this NIR absorption because CTAB molecules partially neutralize the charges from hydroxyl groups, thereby inhibiting water adsorption and reducing NIR reflection. Absorptivity measurements revealed that HSNPs experienced NIR absorption much less than other oxide NPs (Figure S4). However, this can result in intermolecular aggregation, consequently diminishing the printability of HSNPs (middle, Figure 3(b)). To counteract this aggregation, PVP molecules that exert repulsive forces on HSNPs were employed (right, Figure 3(b)).

To achieve a uniform dispersion of HSNPs, it is crucial to determine the appropriate MWs and quantity of PVP molecules attached to their surfaces [45], [46]. We synthesized HSNPs using PVP molecules with varying MWs and subsequently drop-cast them onto silicone substrates. The level of particle dispersion was qualitatively assessed by comparing scanning electron microscopy (SEM) images of the prepared HSNP samples (Figure 3(c)). PVP molecules with lower MWs exhibit insufficient repulsive forces, leading to particle aggregation. Conversely, PVP molecules with excessive MWs shift from functioning as dispersants to binders, resulting in particle clustering. The optimal particle dispersion was achieved using 25 mg of PVP with a MW of 55,000 in a total reaction mass of 37.8 g (the third image on the left in Figure 3(c)).

Based on the established PVP conditions, we uniformly coated HSNPs on silicone substrates using FDM 3D printing (Figure 3(d)) and spray coating (Figure 3(e)) techniques. Both the fabricated samples exhibited ultrawhite and antiglare surfaces. These optical characteristics are a direct result of the scattering characteristics of the HSNPs, as shown in Figure 2. For accurate reflectance measurements, the sample size must exceed the dimensions of the beam spot, which is typically larger than 2 × 2 cm^2^. To ensure the absence of gaps between printed lines, we employed an overlaid printing process. This enhanced the reliability of the reflectance measurements of the 3D-printed samples. For both techniques, pastes containing HSNPs were formulated using PVP molecules with two different MWs in a 1:1 mixing ratio with a concentration of 1 wt% relative to the total solution. The use of PVP under unsuitable conditions resulted in the formation of cracks in the printed sample (Figure S5). In general, the adhesion between the NPs and substrates is not robust. However, in real-world applications such as solar panels, protective cover glass is typically mounted on printed patterns, which mitigates potential adhesion issues. To emulate a setting in which a protective layer is employed, we deposited a 60 nm-thick Al_2_O_3_ layer through atomic layer deposition (ALD) on an HSNP-coated sample (Figure S6(a)). The solar reflectance of the HSNP-coated sample remained practically the same throughout a consecutive 13-day outdoor test, indicating its weather resistance (Figure S6(b)).

### 2.4 Spectrum analysis and outdoor experiments

**Figure 4:**
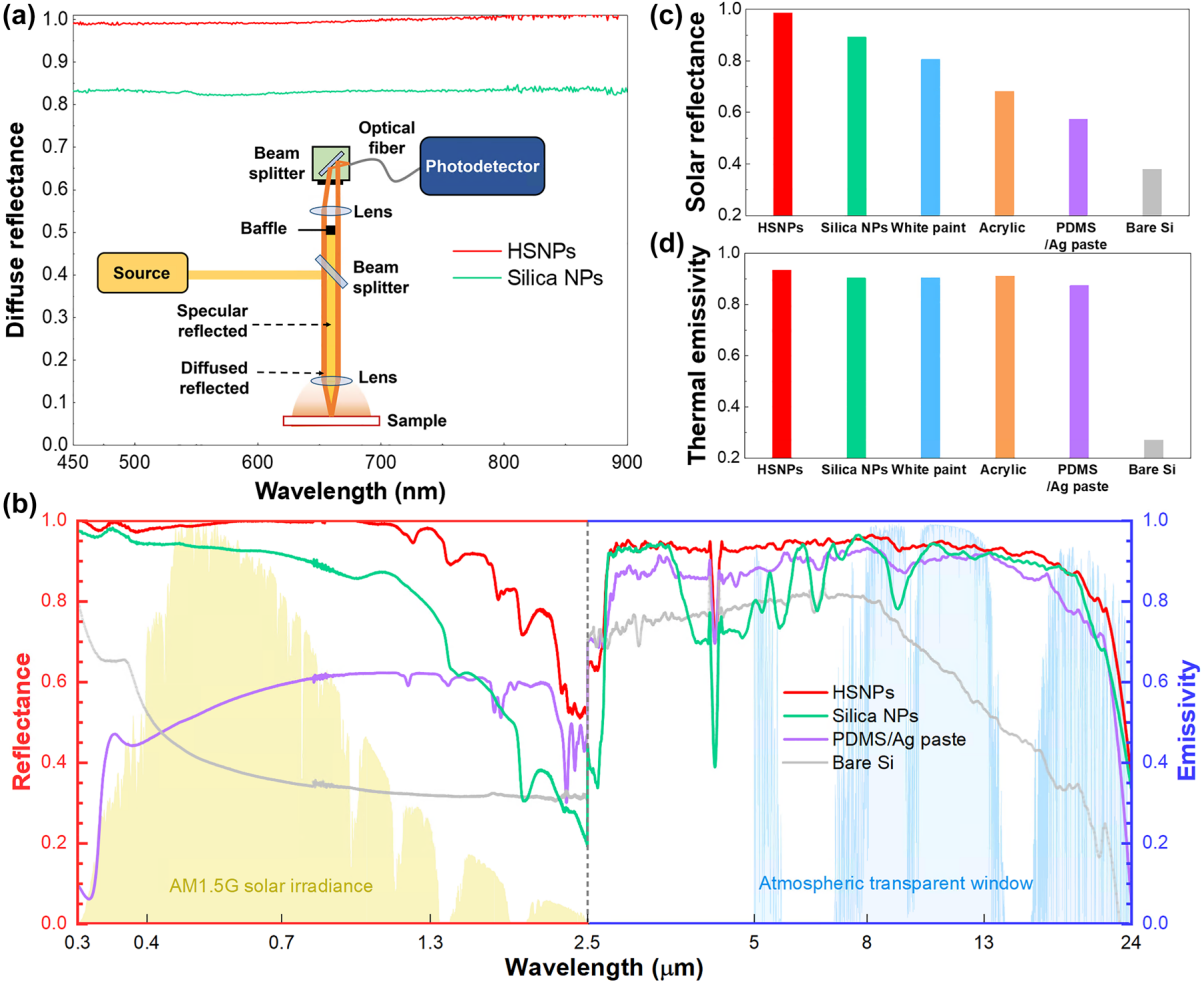
Spectrum analysis of various passive cooling materials. (a) Measured diffuse reflectance spectra of HSNP- and silica NP-coated Si. Each value is normalized by a standard diffuse reflector. Inset: schematic for the setup. (b) Measured reflectance and emissivity values of various passive cooling materials across solar (0.3–2.5 mm) and thermal (8–13 mm) spectra. (c, d) Average solar reflectance (c) and thermal emissivity values (d), based on the data presented in (b).

To support the simulated scattering characteristics shown in Figure 2, we obtained the diffuse reflectance of the synthesized HSNPs and their silica NP counterparts using dark-field microscopy (inset, Figure 4(a)) and angle-dependent reflectance measurements (Figure S7). Notably, the HSNP-coated sample showed an approximate 0.16 increase in average diffuse reflectance compared to the silica NP-coated sample (Figure 4(a)), which is consistent with the simulated result in Figure 2(b) and the higher reflectance observed at various angles. In addition, to verify the antiglare characteristics of the HSNPs, we obtained scattering distributions for both the HSNP- and PDMS on silver paste (PDMS/Ag paste)-coated samples (Figure S8). The HSNPs served as a uniformly diffused surface in stark contrast to the reference silver paste that was characterized by strong specular reflection.

Solar reflectance and thermal emissivity measurements were conducted in the wavelength range of 0.3–24 μm for HSNPs and silica NPs along with various reference materials, including commercial white paint, white acrylic, PDMS/Ag paste, and bare silicone (Figure 4(b) and S9). Within the solar spectrum, the average reflectance of the HSNP-based sample reached approximately 0.98, surpassing those of the silica NP- and white paint-based samples by approximately 0.1 and 0.18, respectively (Figure 4(c)). These findings result from the amplified backward scattering characteristics of individual HSNPs and the effective mitigation of NIR absorption owing to the suppression of both water molecule adsorption and particle aggregation. Although the simulation results supported the superior reflectance of the HSNPs relative to that of the reference silica NPs for all the considered thicknesses, their absolute values were slightly lower than the corresponding experimental data. This discrepancy in reflectance is attributed to various structural factors [46], [47], such as the arrangement, density, and size distribution of the NPs. Our simulation aimed to qualitatively assess the overall reflectance of 3D-packed NPs based on their morphology. While a gradual decline in reflectance for the HSNPs was observed beyond 1.3 μm, the reduced sunlight penetration to the earth’s surface through atmospheric moisture minimized the impact on overall solar reflectance metrics. In comparison, the HSNP-based sample exhibited a modest enhancement in thermal emissivity within the atmospheric transmission window (8–13 μm); its thermal emissivity was 0.93, while other reference samples had similar thermal emissivity values between 0.87 and 0.91. The HSNP-based sample negated reflection caused by phonon–polariton resonance of oxide materials, positioned at 9–11 μm. In general, hollow oxide-shell NPs localized their scattered electric field within the hollow core, which mitigates the excitation of the phonon–polariton resonance of the oxide shell [36]. Note that both spray-coated and 3D-printed HSNPs exhibited nearly the same solar reflectance and emissivity values, which underscores the versatility of our passive cooling material for various applications.

**Figure 5:**
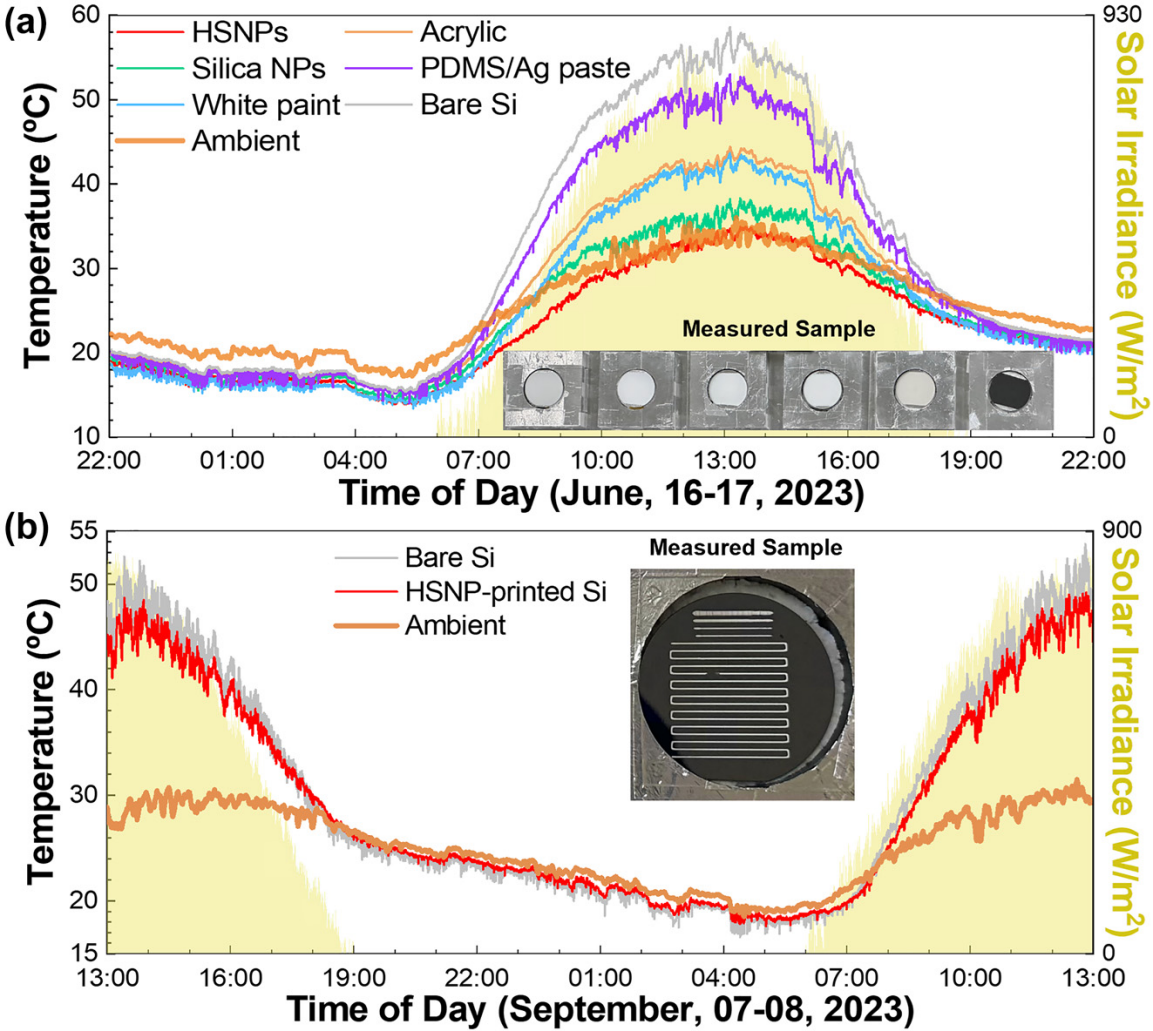
Outdoor cooling experiments. (a) Temporal changes in temperature for the passive cooling materials shown in Figure 3(b). Inset: photographs of the measured samples: HSNP-coated, silica NP-coated, white paint-coated, acrylic, PDMS/Ag paste-coated, and bare silicone substrate samples, from left to right. (b) Temporal changes in the temperature of bare and HSNP-printed (a filling ratio of 10 %) Si substrates.

All-day outdoor cooling experiments were conducted on the same samples (inset of Figure S10(a)). Temporal temperature data were recorded using thermocouples attached to the samples (Figure S10(a)). These samples were positioned on thermally insulating foams and shielded with aluminum Mylar along with a thermally transparent PE film for added protection (Figure S10(b)). The experimental results, `including the solar irradiance power, are shown in Figure 5(a). Details of the wind speed and humidity during the measurement period are shown in Figure S11. The radiative cooling capability is strongly dependent on environmental conditions [1]. In particular, nonradiative heat transfer owing to wind and humidity alters cooling performance. The subambient cooling phenomenon becomes more distinct at lower nonradiative heat transfer coefficients (i.e., low wind speed and humidity), whereas higher nonradiative heat transfer coefficients are beneficial for cooling above ambient temperatures. At the peak solar irradiance (928 W/m^2^), the HSNPs cooled the silicone substrate even lower than the ambient temperature by 2.6 °C. In contrast, the reference silica NPs, PDMS/Ag paste, white paint, and white acrylic increased the temperature by 5.6, 19.6, 10.7, and 11.8 °C, respectively, compared to the ambient temperature. The difference in temperature resulted from their distinct solar reflectance values. The order of the temperatures of the samples followed a reverse order of their solar reflectance (Figure 4(c)). In this context, the HSNP sample exhibited the lowest temperature due to its highest solar reflectance. During the night, the HSNP-coated silicone maintained an average temperature reduction of 3.3 °C compared to the ambient temperature. The other reference samples exhibited almost the same level of cooling performance at night, owing to their similar average emissivity values between 0.87 and 0.93. We prepared an HSNP-printed sample to emulate a real-world scenario in which HSNPs were printed on the top interdigitated electrodes of silicone solar cells. The printed pattern covered approximately 10 % of the overall surface area. Outdoor cooling experiments revealed that the HSNP-printed silicone substrate maintained a temperature approximately 5 °C lower than its reference uncoated sample (Figure 5(b)).

## 3 Conclusions

We synthesized HSNPs using an *in-situ* sol–gel emulsion method under ambient conditions, using PVP as a binder molecule to enhance particle dispersion uniformity. This results in the creation of a subambient passive cooling material applicable to intricate 3D microstructures and surfaces with varying curvatures. Notably, this passive cooling material can be integrated into external microscale electrode configurations, offering the potential for improved energy efficiency in outdoor optoelectronic devices such as solar cells. We highlight that a challenge lies in reducing the linewidth to the standard value (approximately 20 μm) for solar cell electrodes [29]. Further research will prioritize the incorporation of HSNPs into working solar cells to validate the potential efficiency enhancements in real-world settings. Given their advantages, including ambient production capability and enhancement of refractive index contrast within high-refractive-index matrix materials, we anticipate that the developed HSNPs will find applications in cooling various materials, such as polymers, wood, and windows.

## Supplementary Material

Supplementary Material Details
