# Supplementary material for: Three-dimensionally printable hollow silica nanoparticles for subambient passive cooling
Source: Nanophotonics. 2024 Jan 23;13(5):611–20. doi: 10.1515/nanoph-2023-0603 (PMC11501653; doi:10.1515/nanoph-2023-0603)
Supplement: Supplementary file 1 — Supplementary Material Details [file j_nanoph-2023-0603_suppl_001.docx]

***Supporting Information for***

Three-Dimensionally Printable Hollow Silica Nanoparticles for Subambient Passive Cooling

Su-Jin Park^1^, Seok-Beom Seo^1^, Jiyun Shim^2^, Seok Jin Hong^2^, Gumin Kang^3^, Hyungduk Ko^3^, Sunho Jeong^2*^, and Sun-Kyung Kim^1*^

^1^Department of Applied Physics, Kyung Hee University, Gyeonggi-do 17104, Republic of Korea

^2^Department of Advanced Material Engineering for Information & Electronics, Kyung Hee University, Gyeonggi-do 17104, Republic of Korea

^3^Nanophotonics Research Cehter, Korea Institute of Science and Technology, Seoul 02792, Republic of Korea

^1*^S.-K.K. (sunkim@khu.ac.kr)

^2*^S.J. (sjeong@khu.ac.kr)

**This PDF file includes the following sections:**

Methods

Supplement Figures S1–S12

**Methods**

**Integrated ray-wave optics simulation.** To support the measured reflectance spectra for both the 3D-packed HSNPs and silica NPs, integrated ray-wave optics simulations were performed. In the first step, we conducted wave-optics simulations (COMSOL^®^ Multiphysics^TM^ 5.6 wave optics module) on single HSNPs and silica NPs to determine their scattering distributions. Silica was treated as a material with a constant refractive index (*n* = 1.5 and *k* = 0) in the visible wavelength range (400–700 nm). The simulation domain was centered on single NPs with a diameter of 500 nm. For the HSNPs, the silica shell thickness was 70 nm. Our simulations incorporate perfectly matched layers and scattering boundary conditions. Plane waves with visible wavelengths (400–700 nm) and a 10-nm wavelength step were generated. To calculate the reflectance values for both 3D-packed HSNPs and silica NPs, we utilized the scattering distributions obtained from the wave-optics simulations and incorporated them into the models for ray-optics simulations (LightTools® 9.0). Each scattering event was assigned a mean free path of 500 nm, which is equivalent to the diameter of the nanoparticles. A total of 100,000 rays were excited, and the simulations allowed up to 100,000 scattering events. The underlying silicon substrate was characterized by an absorbance of 0.7 and a reflectance of 0.3.

**Synthesis of 3D printable HSNPs.**  HSNPs were synthesized through the ammonia-catalyzed hydrolysis and condensation of TEOS, employing a mixed solvent comprising ethanol and water, with cetyl trimethyl ammonium bromide (CTAB) as the surfactant. The reaction mixture consisted of deionized (DI) water (25.3 g), ethanol (12.0 g), TEOS (470 mg), and CTAB (80 mg). Subsequently, 470 mg of a 25 wt% ammonia solution was added while maintaining continuous stirring at a temperature of 25 °C. Notably, our experimental observations indicated that the diameter of the HSNPs increased linearly when the DI water/ethanol ratio was in the range of 0.3–0.6. This specific range is crucial for the successful formation of the desired hollow morphology. Outside this defined range, the formation of the intended hollow structure is significantly compromised. Five minutes after the initiation of the reaction, 25 mg of PVP with a MW of 55,000 was added. Following a reaction time of 3 h, the resulting products underwent centrifugal washing with DI water twice. All the precursor materials were purchased from Sigma-Aldrich. A schematic of the overall synthesis process is shown in Figure S12. To prepare the 3D printing paste, the HSNPs, which were preserved in DI water, were washed using isopropyl alcohol under centrifugal conditions set at 7000 rpm for 20 min. From the pellet accumulated at the base of the container, an aliquot of 1.8 g was extracted. This aliquot was combined with 1.91 g of ethylene glycol and PVP (20 mg) with MWs of 1,300,000 and 55,000, respectively, in an equimolar ratio of 1:1. The constituents were combined using a Thinky ARE-310 mixer to obtain the desired pastes.

**Spray coating.** Prior to spray coating, the silicon substrate was treated with oxygen plasma using reactive-ion etching equipment for an exposure duration of 10 s. The spray coater consisted of a dispenser (Musashi Engineering ME-5000SP), a motion controller (Musashi Engineering Shot Mini-200Sc equipped with a custom-made heating stage), and a spray valve (Musashi Engineering SV-6). The temperature of the lower stage was maintained at 125 °C throughout the process. Specific spray settings, were applied including a blow pressure of 150 kPa, valve pressure of 423 kPa, and tank pressure of 14 kPa. The spray valve, positioned 12 cm above the wafer, was moved at a controlled speed of 52 mm/s and underwent 120 cycles, each lasting 1 s. For secure anchoring of the substrate, both the upper and lower sections were affixed using polyimide tape.

**3D printing.** For the 3D printing process, a programmable dispenser (Image Master 350PC Smart by Musashi) was used with the following parameters: a nozzle diameter set at 330 μm, pneumatic pressure applied at 60 kPa, and a gap of 350 μm. These settings resulted in a printing velocity of 10 mm/s. The Si substrate was treated with ultraviolet ozone for 1 min to prepare it for printing.

**Optical characterizations.** Solar reflectance measurements of the HSNPs, silica NPs, and other reference materials were conducted using a spectrophotometer (Cary5000) equipped with an integrating sphere. The solar

reflectance ($R_{sol}$) was determined by integrating the reflectance data at the wavelengths of 0.3–2.5 μm and weighting it as per the AM1.5G solar irradiation power profile ($I_{AM1.5G}\left( \lambda\right)$), as defined by the following equation:

| $R_{sol}=\frac{\int_{0.3\mu m}^{2.5\mu m} R\left( \lambda\right) I_{AM1.5G}\left( \lambda\right) d\lambda}{\int_{0.3\mu m}^{2.5\mu m} I_{AM1.5G}\left( \lambda\right) d\lambda}$, | (1) |
| --- | --- |

where $\lambda$ represents the wavelength and $R\left( \lambda\right)$ represents the measured reflectance at each wavelength. To establish baseline correction, a standard diffuse reflector (SRS-99-010, Labsphere) was employed. The data were calibrated by multiplying them with the absolute reflectance spectrum data of the standard diffuse reflector.

The angular reflectance characteristics were obtained using an integrating sphere (CSRM-RTC-060-SL, Labsphere) and a spectrophotometer (Maya 2000 Pro, Ocean Optics). To block the sample mounted on the integrating sphere, an SRS-99-010 reflector was utilized. The samples were positioned at the center of the integrating sphere, and measurements were taken at incident angles ranging from 10° to 50°.

To acquire the thermal radiation spectrum, we employed an FTIR spectrometer (INVENIO R, Bruker) equipped with a gold diffuser-coated integrating sphere (A562-G/Q, Thorlabs) and a DTGS detector. This setup enabled us to obtain reflectance and transmittance spectra within the mid-infrared wavelength range (2.5–24 μm). Emissivity values were determined using the following equation:

| $\varepsilon_{measured}\left( \lambda\right)=1-R_{measured}\left( \lambda\right)-T_{measured}\left( \lambda\right)$, | (2) |
| --- | --- |

where $\varepsilon_{measured}\left( \lambda\right)$ denotes the measured emissivity, $R_{measured}\left( \lambda\right)$ denotes the measured reflectance, and $T_{measured}\left( \lambda\right)$ denotes the measured transmittance.

For dark-field microscopy, we employed an optical microscope (LV100ND, Nikon) equipped with a Maya 2000 detector (Maya 2000 Pro, Ocean Optics) in the dark-field mode. An integrated halogen lamp within the microscope was used as the light source. A 20x objective lens was utilized, and the equipment in episcopic illumination mode quantified the light intensity reflected from the sample surface.

**Outdoor cooling experiments.** The outdoor cooling experiments were conducted on the rooftop of the Department of Physics, Kyung Hee University, Yongin-si, Gyeonggi-do, Republic of Korea, 17104. The measurement setup consisted of an acrylic box coated with an aluminized Mylar film, which minimized the heat exchange with the ground. Each sample was placed inside an acrylic box covered with an aluminated Mylar film, leaving the surface exposed to direct sunlight. A polystyrene thermal insulator was placed on the rear side of each sample holder. To act as a windshield, a low-density polyethylene film, transparent to mid-IR radiation, was wrapped around the setup. To monitor temporal changes in temperature, adhesive thermocouples (SA1-K-72-SC, Omega Engineering) were affixed to the silicon substrates. These temperature readings were recorded at 10-s intervals using a data logger (RDXL12SD, Omega Engineering). Both HSNPs and silica NPs were prepared using the previously described spray coating method. A white paint sample was created by spin-coating a commercial paint (YG6111, Momentive) onto a silicon substrate at 500 rpm for 120 s. The PDMS/Ag paste sample was produced by sequentially spin-coating silver paste (Elcoat p-100, CANS) and PDMS under the same conditions, specifically at 500 rpm for 120 s. Silica NPs, PDMS, and the curing agent were purchased from Sigma-Aldrich.

**
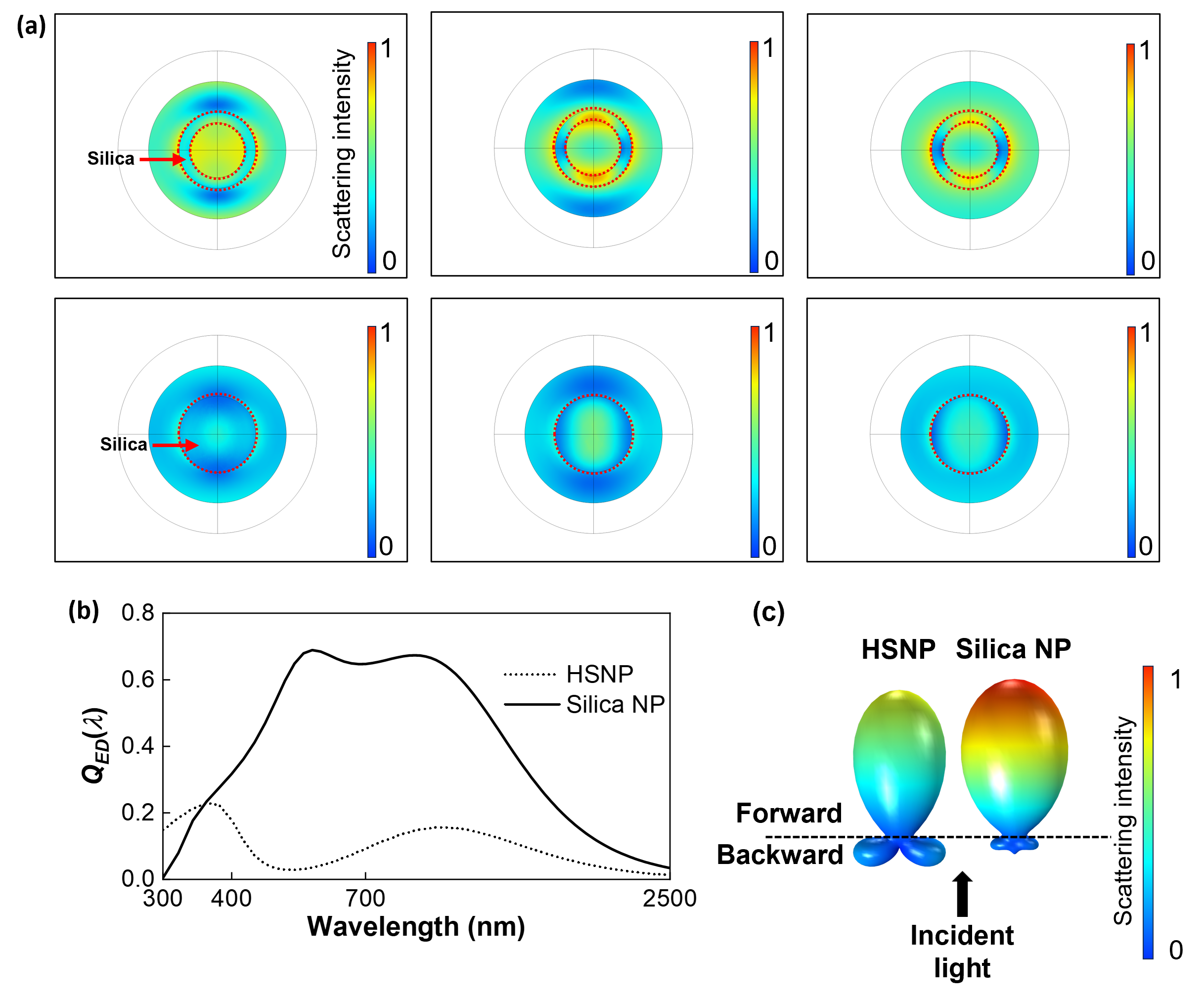
**

**Figure S1. Scattering analysis of single HSNP and silica NP.** (a) Cross-sectional scattered electric fields of the HSNPs (top panels) and silica NPs (bottom panels), obtained at three sampled visible wavelengths: 450, 550, and 650 nm (from left to right). The red dashed lines represent the boundary of each NP. (b) Calculated scattering efficiencies (*Q*_ED_) of HSNPs and silica NPs with the same diameter of 500 nm. Only electric dipole contribution was considered in the scattering calculation. (c) 3D angular scattering distributions of both NPs.


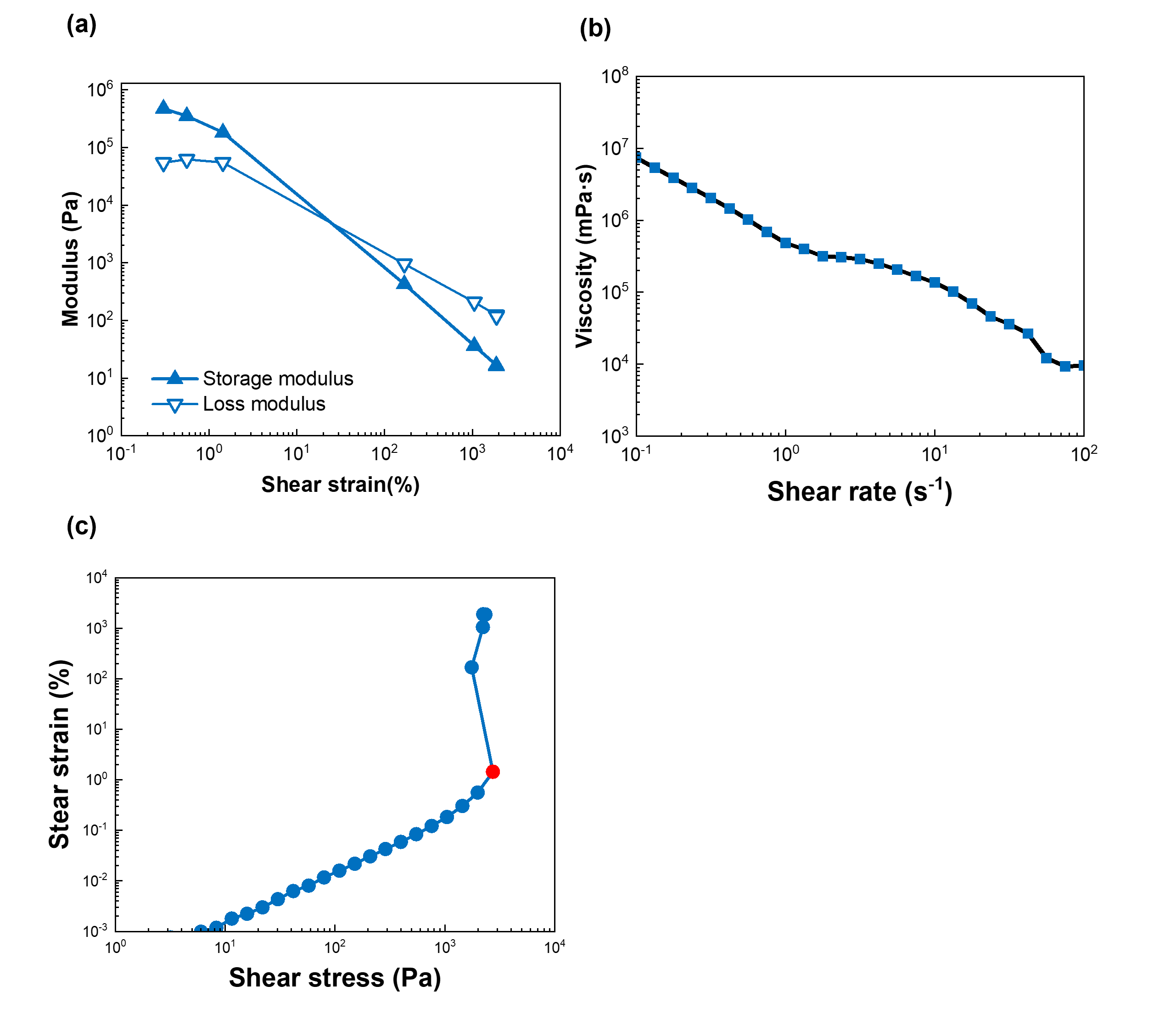


**Figure S2. Rheology analysis of 3D printing paste.** (a) Storage (G′) and loss (G′′) moduli of the paste. (b) Apparent viscosity of the paste as a function of the shear rate applied in a steady-shear experiment. (c) Shear strain of the paste when subjected to an increasing shear stress in a quasi-static experiment. The red full circle indicates the apparent yield stress of the paste.

**
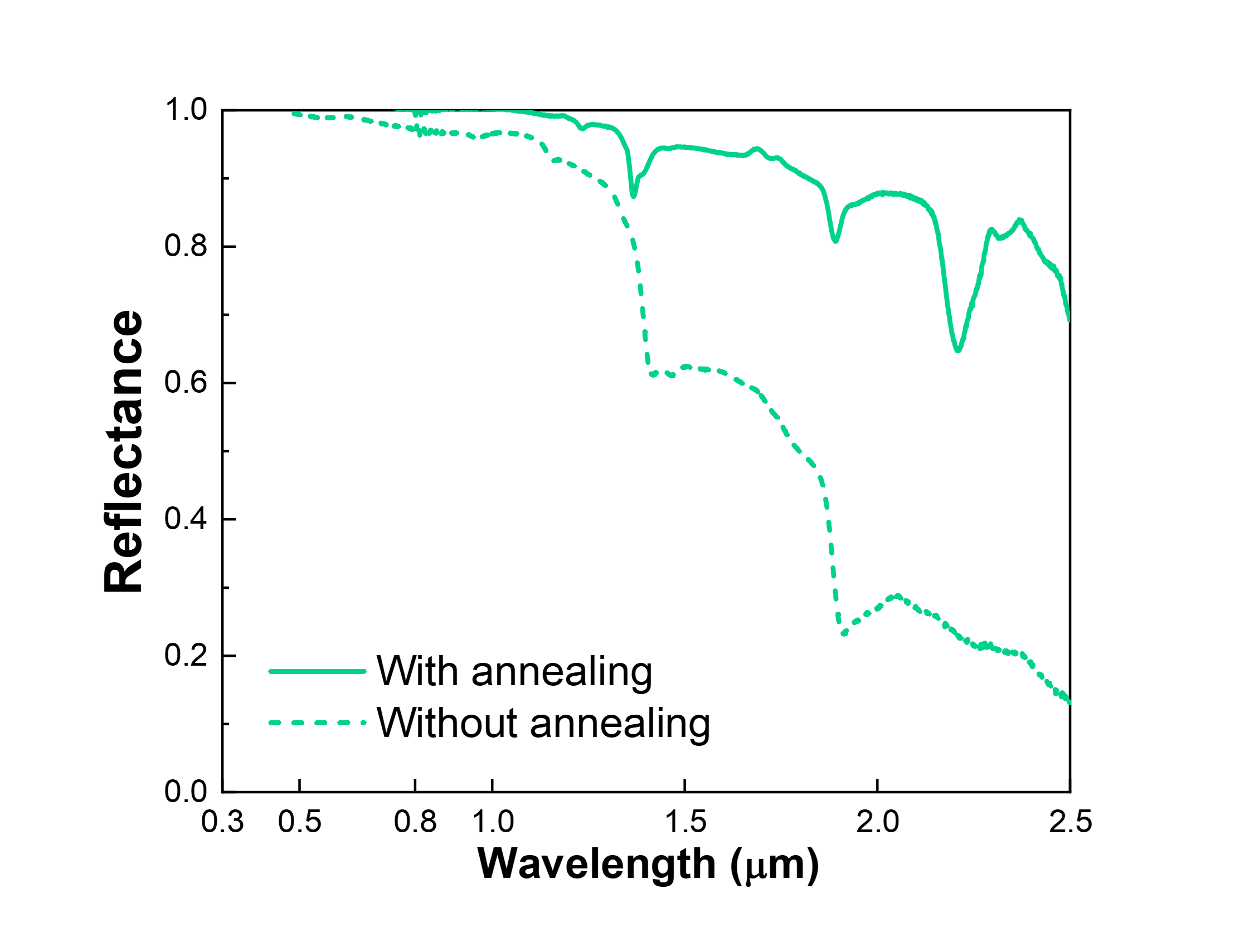
**

**Figure S3.** **Effect of thermal annealing.** Reflectance spectra of silica NPs before and after thermal annealing at 800 $℃$ for 60 min. After the thermal annealing, the hydroxyl functional groups of silica were eliminated, leading to a decrease in the absorptivity within the near-infrared spectrum. This experiment reveals that the near-infrared absorption of pristine silica NPs results from their water adsorption.

**
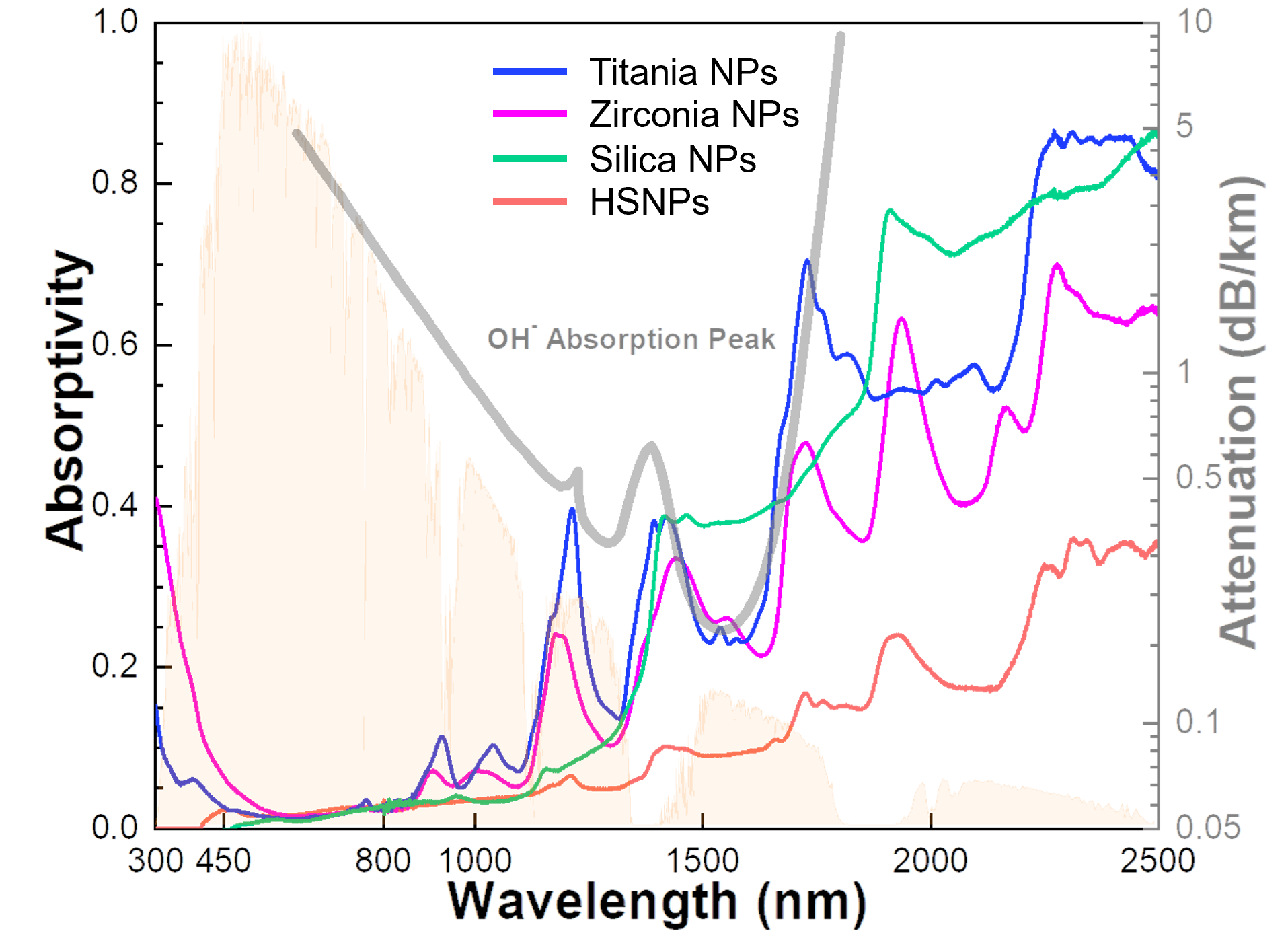
**

**Figure S4. NIR absorption of various oxide NPs**. Measured absorptivity spectra of HSNPs and silica NPs, along with other reference oxide NPs.


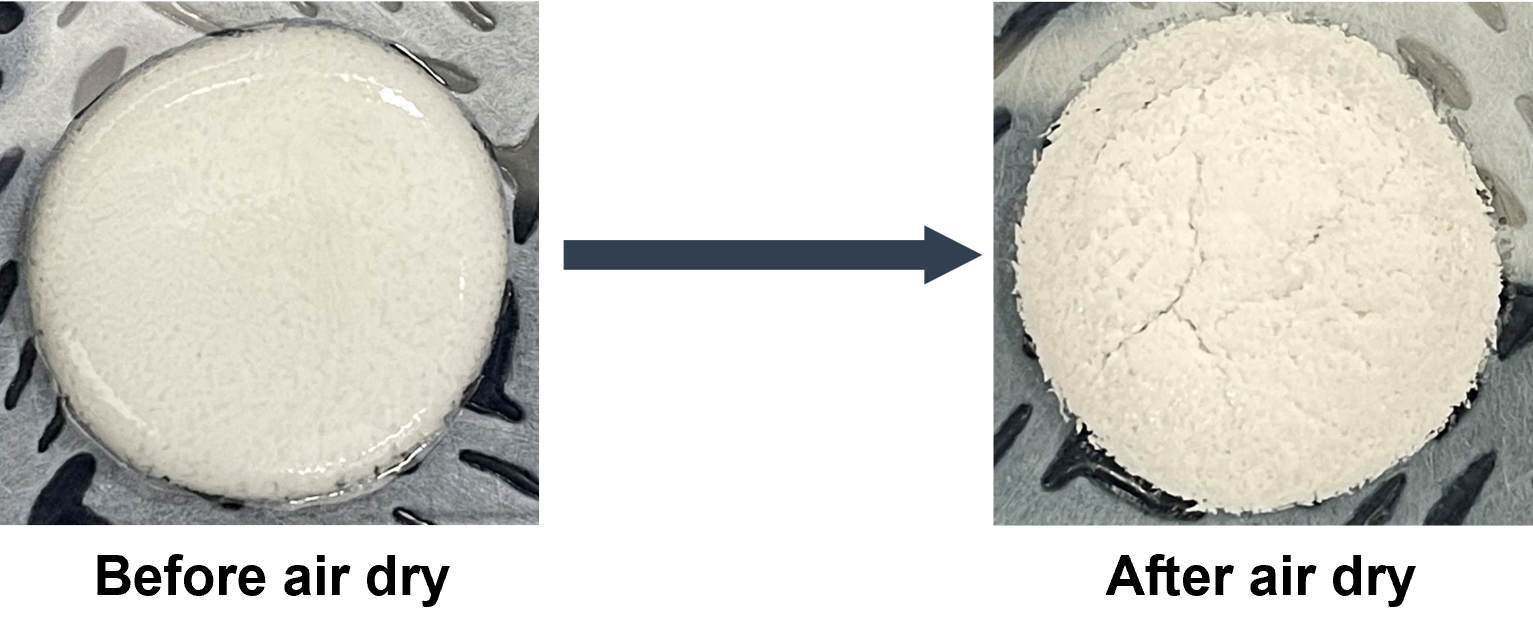


**Figure S5. HSNPs with PVP under inappropriate conditions.** Photographs of a HSNP-printed sample before and after air drying.


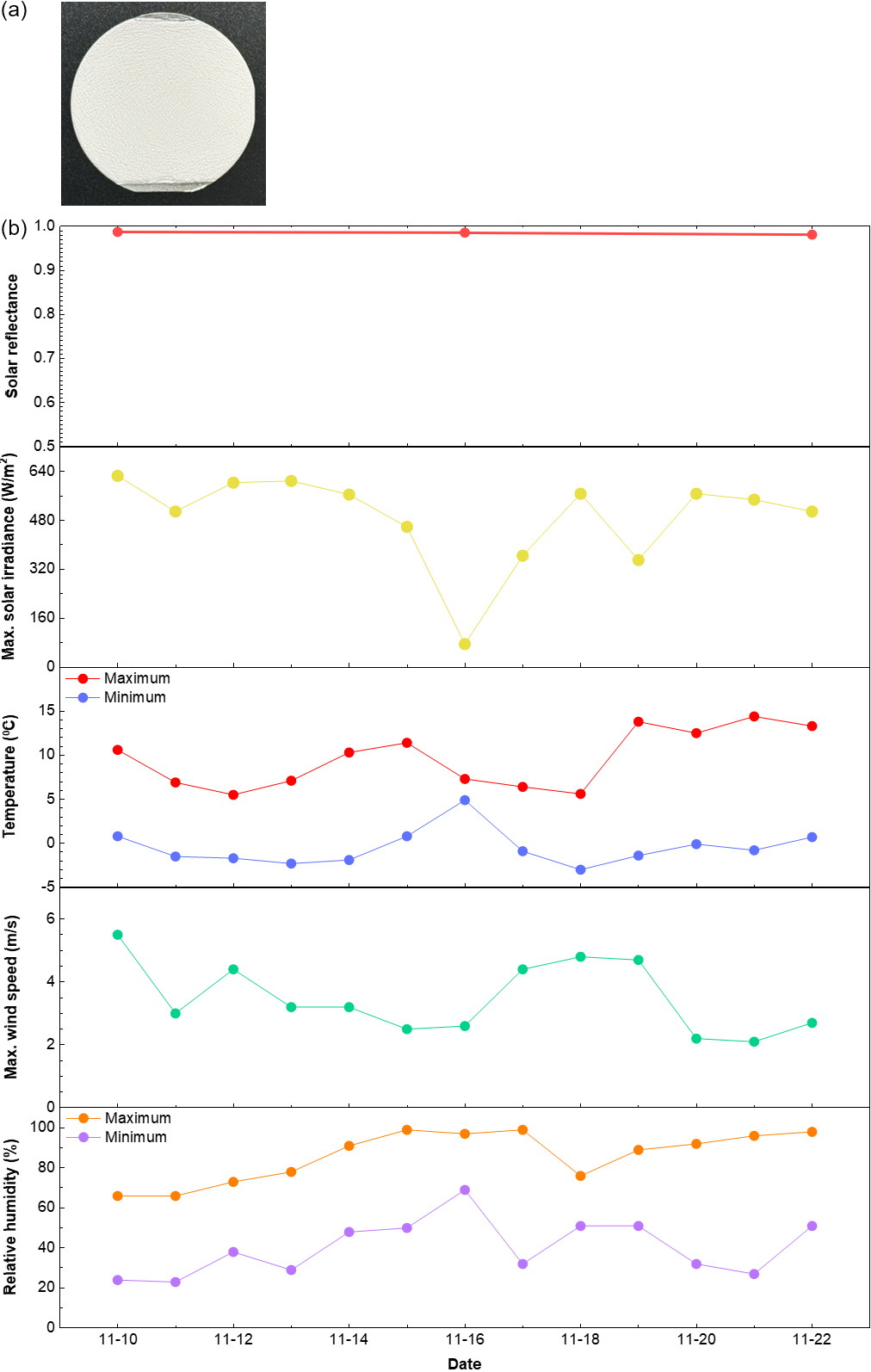
**Figure S6. Weather resistance test of HSNPs.** (a) Photographic image of a fabricated HSNP-coated sample. A 60 nm-thick Al_2_O_3_ layer was deposited on the sample through ALD. (b) Measured temporal changes in solar reflectance for the fabricated HSNP-coated sample during outdoor testing.

**
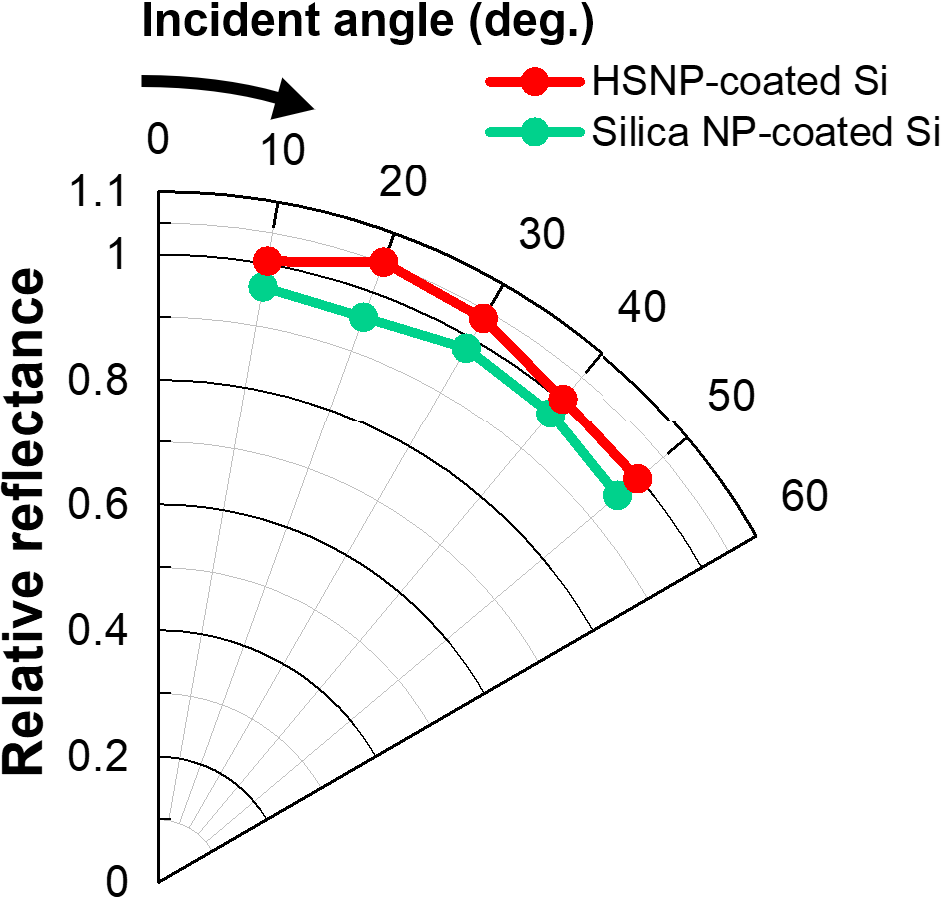
**

**Figure S7. Angular reflectance spectra.** Measured angular reflectance of HSNP-, and silica NP-coated silicon substrates, obtained at the wavelength of peak solar intensity (550 nm). The HSNP-coated sample exhibited higher reflectance compared to that of the silica NP-coated sample for all the considered angles.


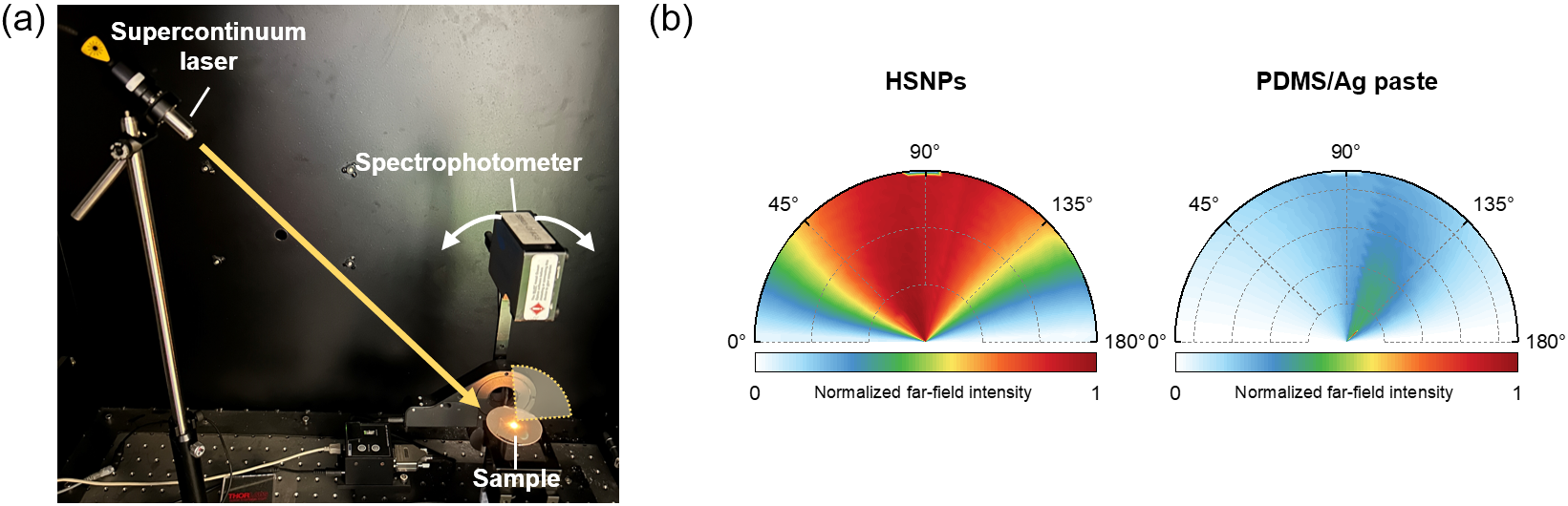


**Figure S8. Measurement of scattering distributions.** (a) Photographic image of the scattering measurement setup. The incident angle of a laser source is 45˚. (b) Measured scattering distributions for both HSNP- and PDMS/Ag paste-coated samples. The HSNPs exhibited uniformly spread scattering distributions, leading to an antiglare effect.

**
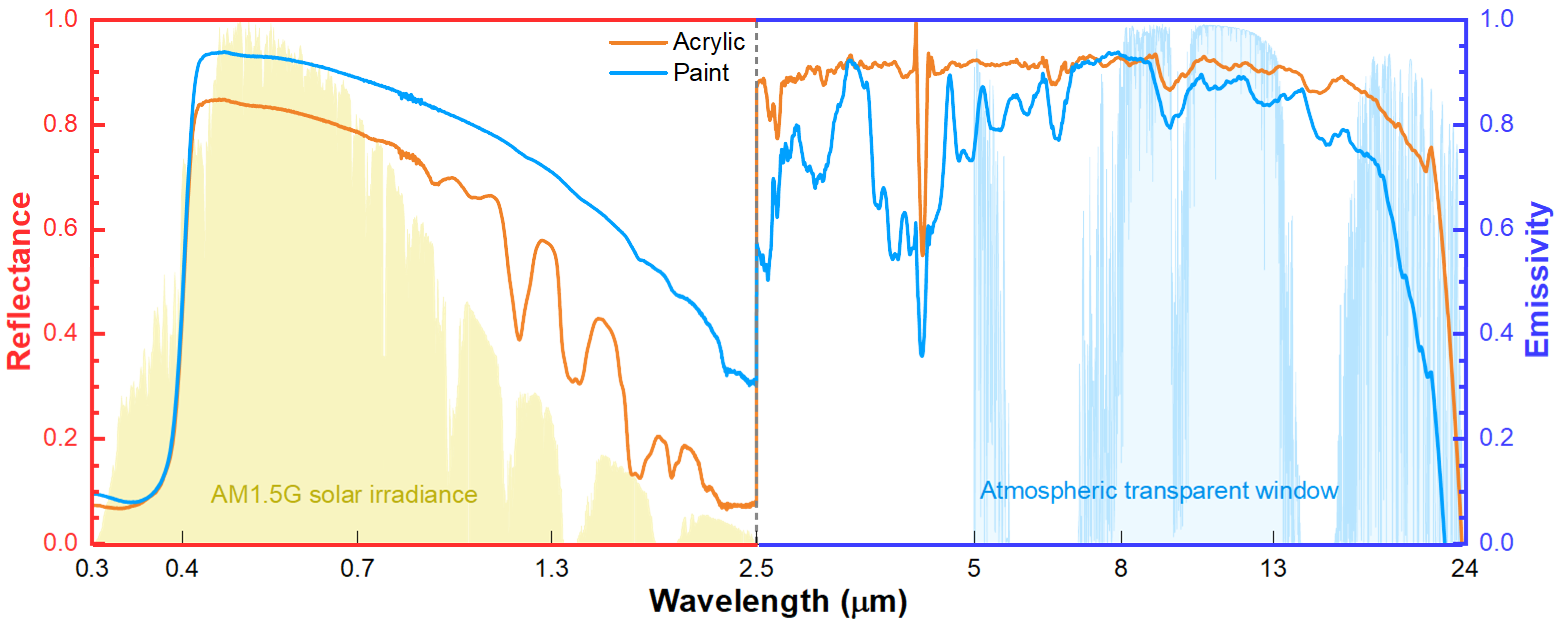
**

**Figure S9. Optical characteristics of commercial white samples.** Measured reflectance and emissivity spectra (0.3–24 μm) of commercial white samples (acrylic and white paint).


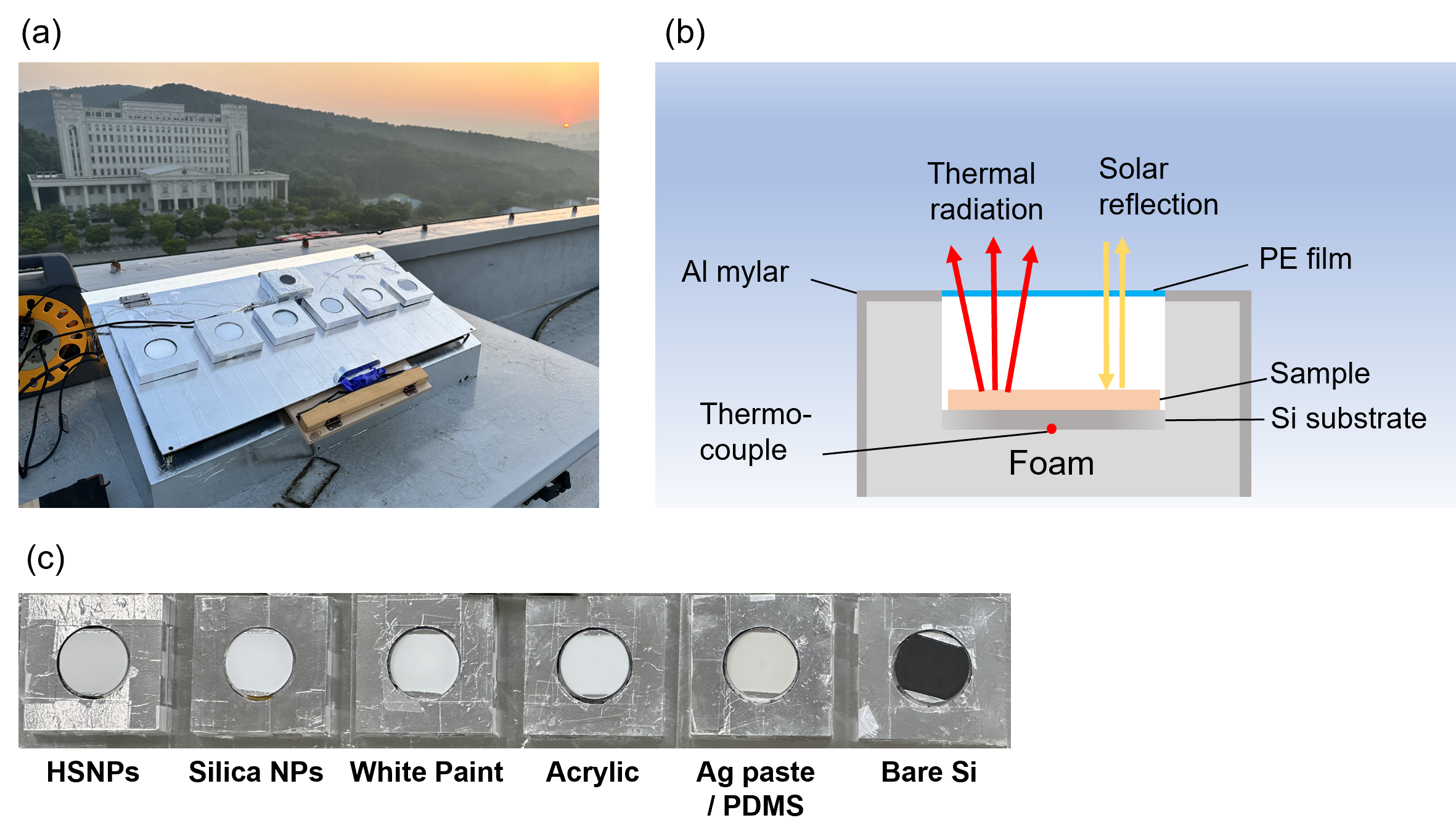


**Figure S10. Setup for outdoor cooling experiments.** Photograph (a) and its schematic (b) for the outdoor cooling experiments shown in Figure 5.


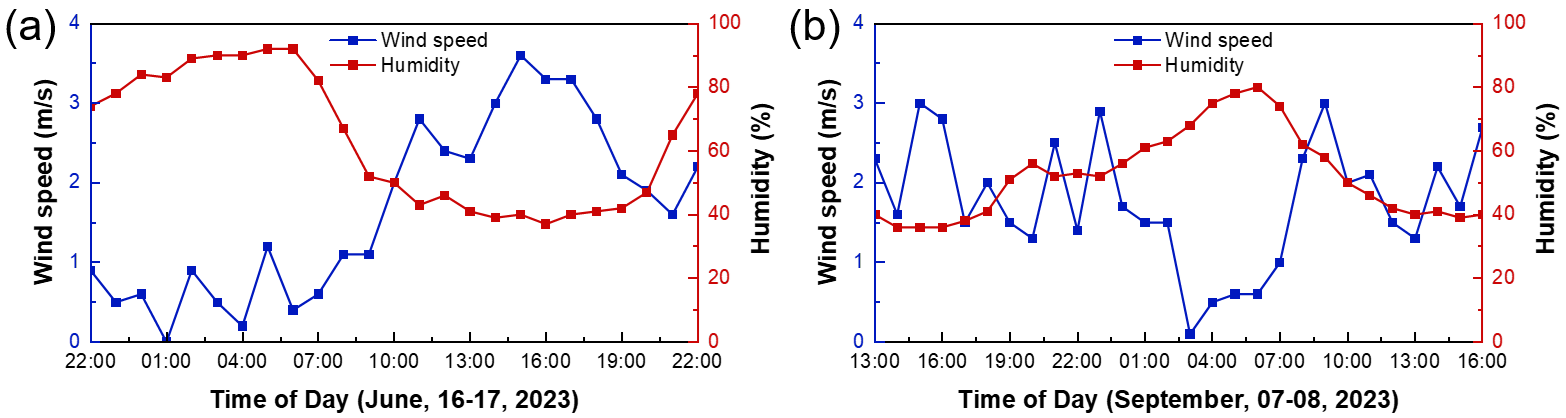


**Figure S11. Other environmental factors during the measurement period.** Temporal changes in humidity and wind speed during outdoor cooling experiments on (a) June, 16–17, 2023, depicted in Figure 5(a) and (b) September, 07–08, 2023 depicted in Figure 5(b).

**
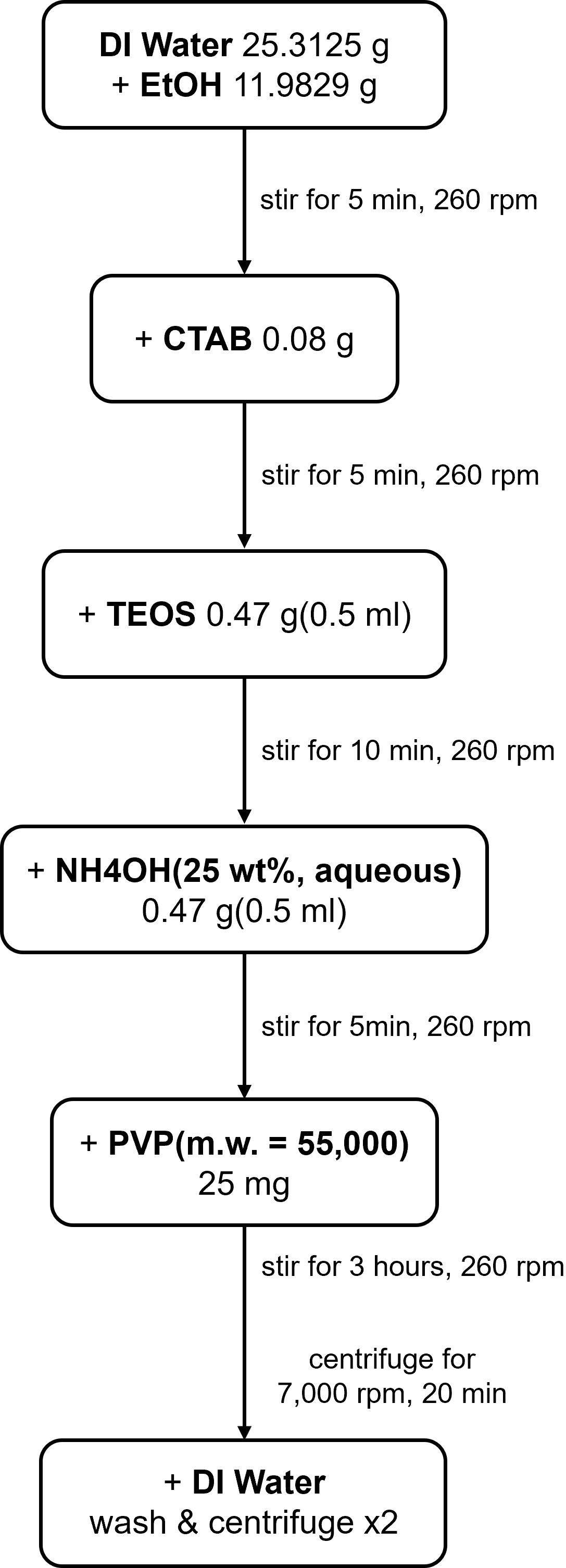
**

**Figure S12. Schematic of overall synthesis process of HSNPs.** Detailed information of the synthesis.
